# Potential of Nb_2_O_5_ as a Catalyst in Biodiesel Production: A Study with Different Feedstock

**DOI:** 10.3390/molecules30051075

**Published:** 2025-02-26

**Authors:** Helder de Lucena Pereira, Adriano Lima da Silva, Carlos Bruno Barreto Luna, Joyce Salviano Barros de Figueiredo, Simoni Margareti Plentz Meneghetti, Ana Cristina Figueiredo de Melo Costa

**Affiliations:** 1 Laboratory of Ceramic Materials Synthesis, Federal University of Campina Grande, 882 Aprígio Veloso Street—Bodocongó, Campina Grande 58429-900, PB, Brazil; adrianolimadasilva@hotmail.com (A.L.d.S.); joyce.barros24@hotmail.com (J.S.B.d.F.); ana.figueiredo@professor.ufcg.edu.br (A.C.F.d.M.C.); 2 Polymer Processing Laboratory, Federal University of Campina Grande, Unidade Acadêmica de Engenharia de Materiais, Av. Aprígio Veloso, 882, Campina Grande 58429-900, PB, Brazil; 3 Catalysis and Chemical Reactivity Group (GCAR), Chemistry Department, Federal University of Alagoas, Maceió 57072-970, AL, Brazil; simoni.plentz@gmail.com

**Keywords:** Nb_2_O_5_ catalyst, biodiesel synthesis, transesterification, methyl route, renewable feedstocks

## Abstract

The objective of this study was to evaluate the catalytic performance of commercial Nb_2_O_5_, supplied by CBMM, in the production of biodiesel by transesterification and esterification, using different feedstocks (soybean, corn, sunflower, and waste oils) and both methyl and ethyl routes. For this, the catalyst was characterized in terms of its crystal structure by X-ray diffraction (XRD), specific surface area using the Brunauer–Emmett–Teller (BET) technique, thermal stability by thermogravimetric analysis (TGA), morphology by scanning electron microscopy (SEM), acidity by ammonia desorption at programmed temperature (TPD-NH_3_), and catalytic activity by gas chromatography. The results from the structural analyses indicated that Nb_2_O_5_ has a single monoclinic phase and a morphology consisting of irregular agglomerates. The specific surface area was 1.3 m^2^/g, and its density was 4.639 g/cm^3^. The thermogravimetric analysis showed that the material has thermal stability, maintaining its structural integrity up to temperatures as high as 1000 °C. The total acidity reached 301 μmol NH_3_/g, indicating the presence of Brønsted and Lewis acidic sites. In catalytic tests, Nb_2_O_5_ showed higher efficiency in the methyl route, achieving an initial conversion of 96.43% in esters with soybean oil, outperforming other feedstocks. However, catalyst reuse over five cycles revealed a progressive decrease in catalytic activity, possibly due to blocking active sites by adsorbed products, as confirmed by FTIR and XRD analyses conducted on the catalyst. Despite decreased activity after the cycles, the catalyst maintained its crystal structure, indicating structural stability. These results demonstrate the potential of Nb_2_O_5_ as a heterogeneous catalyst for biodiesel production, particularly with the methyl route and high-quality oils. This study highlights the relevance of Nb_2_O_5_ in biodiesel synthesis, contributing to sustainable practices and technological advancement in the renewable energy sector.

## 1. Introduction

Niobium oxide is one of the most studied transition metal oxides, standing out for its crystalline [1], electrical [2], optical [3], and thermal [4] properties. Its different oxidation states can be controlled through deposition and annealing, allowing for various applications. While NbO exhibits metallic behavior, NbO_2_ and Nb_2_O_5_ show semiconductor and insulating properties [5].

Niobium pentoxide (Nb_2_O_5_) has attracted scientific and technological interest due to its high surface area, mechanical strength, acidic sites, melting point, and thermal stability [6]. The crystal structure of Nb_2_O_5_ depends on the preparation conditions, with the amorphous material being converted, upon increasing temperature, into three distinct phases: pseudohexagonal (TT-Nb_2_O_5_), orthorhombic (T-Nb_2_O_5_), and monoclinic (H-Nb_2_O_5_) [7]. Classified as an n-type semiconductor, this oxide offers potential applications in various fields such as gas detection [8], optical sensors [3], dye-sensitized solar cells [9], energy storage systems [10], catalysis [11], and biodiesel [12,13].

Niobium oxide and its compounds play multiple catalytic roles, acting as promoter supports and exhibiting redox and acidic properties. The firm acidity, stemming from Brønsted and Lewis sites, combined with the water resistance of the Lewis acid sites, contributes to its effectiveness in various catalytic processes [7,14]. For this reason, niobium-based materials have been used as catalysts in alcohol dehydration reactions [15], oxidation [16], photocatalysis [17], isomerization [18], acetalization [19], and esterification [20]. Despite advances in research, it has not yet been fully clarified which crystal phase of Nb_2_O_5_ exhibits the most efficient catalytic performance, possibly due to the complexity of interactions between the crystal structure, active sites, substrate, and reaction conditions.

Biodiesel, long-chain carboxylic acid esters derived from renewable lipids, is attractive due to its ability to significantly reduce pollutant emissions in diesel engines [21]. Its production can be carried out using various lipid sources, catalysts, and technologies, with homogeneous alkaline transesterification being the most widely used at the industrial scale due to its low cost and mild conditions. However, the homogeneous process generates soap and emulsions when the feedstock contains a significant amount of moisture or free fatty acids (FFAs). Moisture can cause the hydrolysis of triglycerides, releasing FFAs that, by reacting with the cations of the homogeneous catalyst, form soap and promote the formation of emulsions, complicating the purification and reuse of the catalyst used [22].

Alternatively, heterogeneous catalysis offers advantages such as easier separation, reusable catalyst, and greater tolerance to carboxylic acids [23,24]. The use of catalysts to promote the transesterification reaction of vegetable oils with alcohols in biodiesel production has been the subject of recent studies by researchers [25,26,27,28,29].

The use of biodiesel blends with petroleum diesel (B20) is a well-established practice in many countries around the world. However, reducing production costs, carefully selecting feedstocks, and defining efficient catalysts for biodiesel synthesis continue to be significant technical and economic challenges in this process [30]. This work innovates by exploring the potential of Nb_2_O_5_ as a catalyst in biodiesel production, a scarcely investigated application with growing relevance. This study not only fills a gap in the scientific literature regarding the use of niobium but also reinforces its versatility and importance in developing alternative and efficient catalysts for sustainable biofuel production processes. The use of commercially available niobium-based materials is particularly relevant, as they offer a cost-effective alternative and remain underexplored in scientific studies.

Moreover, the global demand for niobium currently accounts for less than 0.5% of the known reserves, highlighting the importance of promoting research related to this material. This incentive is particularly significant for Brazil, which holds over 98% of the world’s reserves and is a leader in production and export, with the potential to add greater economic and technological value to this strategic resource [31,32].

Table 1 presents a summary of studies related to the use of niobium as a heterogeneous catalyst in biodiesel production.

Table 1 presents scientific data on applying niobium-based (Nb_2_O_5_) catalysts in biodiesel production processes. Studies involving Nb_2_O_5_ in different configurations and combinations with other oxides or support materials show biodiesel conversion ranging from 70% to 99.2% under various reaction conditions and using different feedstocks.

The application of Nb_2_O_5_/SO_4_ for palm oil stands out, with a conversion of 99.2%, highlighting niobium’s ability to act as a robust catalyst in esterification and transesterification reactions under various conditions. Another promising example is 30MoO_3_/Nb_2_O_5_, which achieved a 94.3% conversion using waste oil, an economically attractive resource.

Although the results are promising, the literature still lacks systematic studies exploring the potential of Nb_2_O_5_ specifically for large-scale biodiesel production. Many of the studies presented use classical synthesis routes, such as impregnation and calcination, with few examples involving innovative methods, such as combustion synthesis or catalyst characteristics after reuse, which could offer advantages in cost reduction and operational simplicity.

This study aims to investigate the catalytic behavior of commercial niobium-based materials in biodiesel production from feedstocks such as soybean, waste oil, corn, and sunflower under conditions of acid heterogeneous catalysis. The influences of experimental variables, such as alcohol type and feedstock, were evaluated, correlating the catalytic results with the physicochemical properties of the materials used. Additionally, the effects of FFA presence in the used catalyst were analyzed, as was the feasibility of its reuse.

## 2. Results and Discussion

### 2.1. Catalyst Characterization

**Figure 1:**
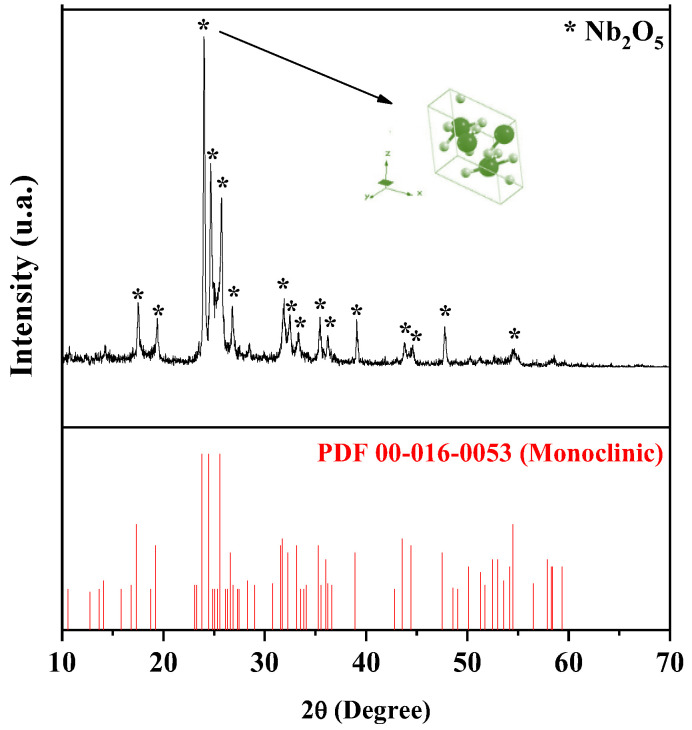
X-ray diffraction pattern for Nb_2_O_5_.

Figure 1 illustrates the X-ray diffraction (XRD) pattern obtained for Nb_2_O_5_, analyzed without any prior treatment, using a scan range of 2θ between 10° and 70°.

Based on the analysis of the diffractogram in Figure 1, it is observed that the material consists of a single crystalline phase of niobium pentoxide with a monoclinic structure, identified by the PDF 00-016-0053 diffraction pattern. Additionally, a crystallinity of 76.7% was determined, indicating a significant predominance of ordered regions within the material. The XRD pattern exhibited well-defined diffraction peaks with high intensity at 2θ values of 17.48°, 19.36°, 23.94°, 24.63°, 25.73°, 26.81°, 31.88°, 32.48°, 33.37°, 35.46°, 36.26°, 39.14°, 43.70°, 44.60°, 47.78°, and 54.52°. These peaks are associated with the characteristic crystal planes of monoclinic Nb_2_O_5_, identified as (−4 0 1), (−1 0 4), (−4 0 5), (−1 0 5), (−6 0 2), (−3 1 1), (−5 1 3), (−5 1 1), (2 1 5), (−6 1 4), (7 0 7), (−6 1 6), (−10 0 3), (6 0 4), (0 2 0), and (−11 1 7).

The symmetry of H-Nb_2_O_5_ is higher than that of the TT-Nb_2_O_5_ and T-Nb_2_O_5_ polymorphs. H-Nb_2_O_5_ is formed after annealing at temperatures above 900 °C and is considered the most stable phase of Nb_2_O_5_. In this structure, each niobium atom is surrounded by six oxygen atoms, forming NbO_6_ octahedral units. The monoclinic lattice of H-Nb_2_O_5_ consists of 3 × 4 and 3 × 5 blocks, where NbO_6_ octahedra are interconnected through edge-sharing. Within each block, the NbO_6_ units are further linked by corner-sharing, creating a stable three-dimensional network [2,38,39,40].

The monoclinic phase of Nb_2_O_5_ can be obtained through various synthesis methods reported in the literature. Recent studies highlight calcination processes [41], high-temperature solid–state reactions [42], sol–gel methods [43], and solvothermal synthesis followed by thermal treatment [44]. Cheng and Athukoralalage [45] proposed a synthesis of Nb_2_O_5_ using cellulose nanocrystals (CNCs) and cellulose nanofibers (CNFs). The CNCs and CNFs were functionalized with poly(2-(dimethylamino)ethyl methacrylate) (PDMAEMA) and then complexed with ammonium oxalate and hydrated niobium (V) through electrostatic interaction. After thermal treatment, orthorhombic Nb_2_O_5_ was initially obtained, and to reach the monoclinic phase, it was necessary to raise the temperature to 1100 °C and extend the heating time from 2 to 10 h.

**Figure 2:**
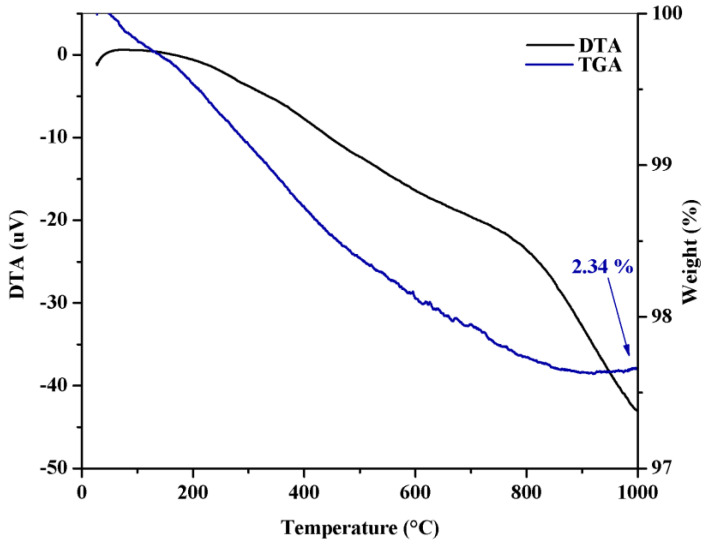
TGA/DTA curves.

Figure 2 illustrates the TGA/DTA curves of Nb_2_O_5_.

The thermal analysis revealed a total mass loss of approximately 2.34% in the temperature range between 49 and 1000 °C, attributed to the removal of both surface and structural water. No significant thermal events were observed that would indicate phase transformations or degradation in the sample, reinforcing the material’s high thermal stability up to its melting point, around 1450 °C, as discussed by da Cruz and Volnistem [46] and Vinodhini and Xavier [47]. These results are consistent with the patterns obtained by X-ray diffraction, which indicate a well-defined single monoclinic phase, further reinforcing the material’s structural stability at temperatures near 1000 °C.

Table 2 presents the values from the semiquantitative analysis of the oxides in the Nb_2_O_5_ catalyst determined by X-ray fluorescence (EDX).

Based on Table 3, the chemical analysis performed by EDX for the Nb_2_O_5_ catalyst revealed that the significant oxide present is NbO, representing 96.71% of the total composition. Other detected oxides include SiO_2_ (2.95%), Fe_2_O_3_ (0.14%), and traces of other oxides, totaling 0.2%. These results confirm that niobium oxide is the predominant component in the catalyst, indicating the high purity of the provided material, while the presence of secondary oxides such as SiO_2_ and Fe_2_O_3_ may be associated with impurities inherent in the raw materials used or the sample preparation process.

**Figure 3:**
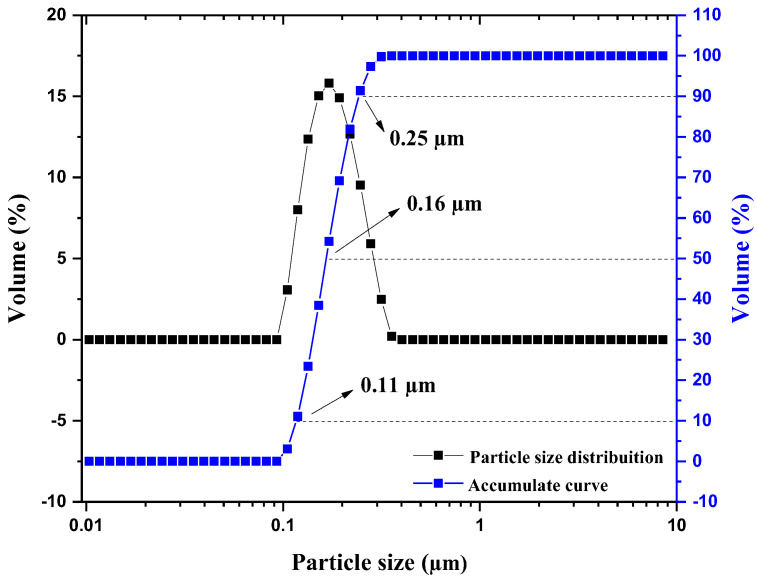
Granulometric distribution of the Nb_2_O_5_ catalyst.

Figure 3 illustrates the granulometric analysis of the Nb_2_O_5_ catalyst, showing the distribution of the equivalent hydrodynamic diameters of the particles relative to the accumulated volume.

The analysis of the granulometric distribution curve of the Nb_2_O_5_ catalyst reveals a unimodal distribution, with particles predominantly in the submicrometric range. The accumulated curve shows a narrow distribution, with 10% of the particles having sizes equal to or smaller than 0.11 µm (D10), 50% with sizes smaller than 0.16 µm (D50), and 90% below 0.25 µm (D90). The average particle diameter of 0.31 µm indicates a high uniformity in particle sizes. Most particles (over 90%) are concentrated within a range very close to the average diameter, with few particles significantly larger or smaller. The average particle size is comparable to the values reported in the literature for pure Nb_2_O_5_ [17].

**Figure 4:**
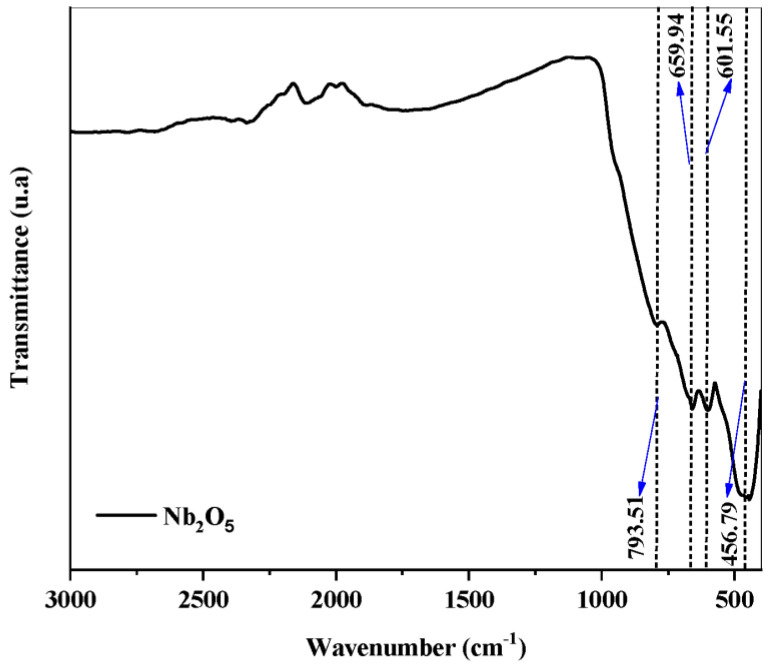
FTIR spectrum of the Nb_2_O_5_ catalyst.

Figure 4 illustrates the vibrational spectrum of the Nb_2_O_5_ catalyst obtained by Fourier transform infrared spectroscopy (FTIR).

The leading absorption bands between 450 and 800 cm^−1^ can be attributed to the characteristic vibrational modes of Nb_2_O_5_. Specifically, the absorptions observed at 601.55 and 793.51 cm^−1^ are associated with the stretching vibrations of the Nb-O-Nb bridge and the stretching vibrations of the Nb=O bond, confirming the presence and structural organization of niobium oxide [3,48].

**Figure 5:**
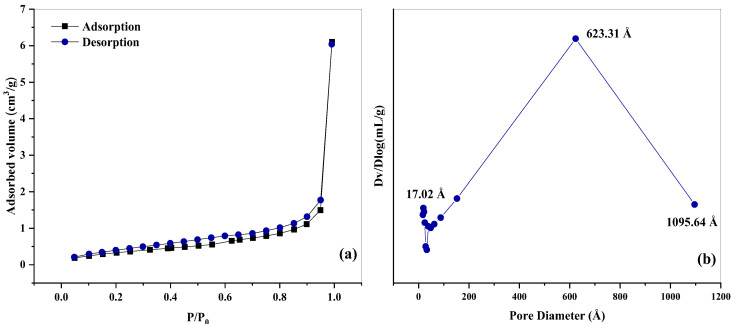
Nitrogen adsorption/desorption isotherms (**a**) and pore size distribution of Nb_2_O_5_ (**b**).

The nitrogen adsorption/desorption isotherms and the pore size distribution for the Nb_2_O_5_ catalyst are illustrated in Figure 5a,b.

According to the Brunauer–Emmett–Teller classification, the isotherms obtained for Nb_2_O_5_ correspond to type IV. The hysteresis loops observed at high relative pressures (0.11 <P/P₀ < 0.95) indicate the occurrence of capillary condensation of gases in the mesopores. The hysteresis loops associated with Nb_2_O_5_ ital correspond to type H3, characteristic of the slit-like pores in the material’s structure. This type of hysteresis is frequently found in solids composed of aggregates or agglomerates of particles, where pore formation results from structural arrangements of plate-like particles or particles with sharp edges resembling cubes [49,50].

The pore size distribution, determined by the BJH method, revealed a wide range of diameters, varying from 17.01 to 1095.64 Å, with a predominant volumetric contribution from pores with an average diameter of 623 Å, highlighting the significant presence of macropores. At the same time, essential contributions from smaller pores below 200 Å were identified, indicating a notable fraction of mesopores. These results are consistent with data reported in the literature for Nb_2_O_5_-based materials [51,52,53].

Table 3 presents the textural and acidic properties of Nb_2_O_5_.

Table 3 presents the values of specific surface area and pore volume for the Nb_2_O_5_ catalyst. The specific surface area of 1.30 m^2^/g reflects a relatively limited surface area. The pore volume of only 0.01 cm^3^/g suggests a predominantly compact structure with low total porosity, a characteristic likely associated with sintering at high temperatures [35,54]. The catalyst’s density, determined as 4.64 g/cm^3^, reflects good compaction of the material, a characteristic that may influence its stability and efficiency in industrial applications. This value is in line with densities reported for niobium oxides, such as 4.6 g/cm^3^ for Nb_2_O_5_, 5.9 g/cm^3^ for NbO_2_, and 7.3 g/cm^3^ for NbO, while the density of metallic niobium is 8.6 g/cm^3^ [55]. The total surface acidity value of 301.00 μmoles NH_3_/m^2^ and 231.01 μmoles NH_3_/m^2^ per unit area indicates a high density of acidic sites in the material. These values within the range are related in the literature [15,56].

**Figure 6:**
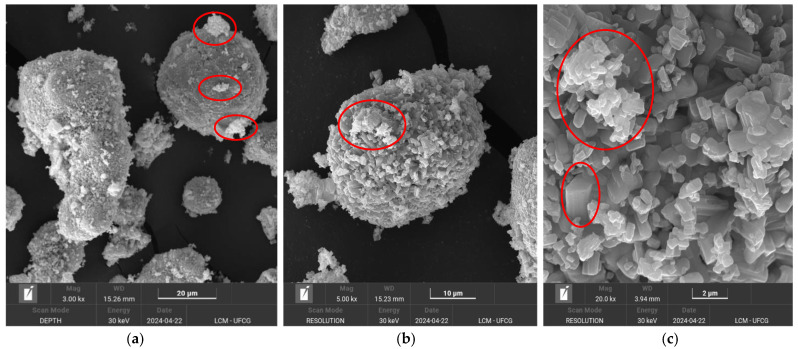
Morphologies obtained by scanning electron microscopy (SEM) for Nb_2_O_5_ were analyzed at magnifications of 3000× (**a**), 5000× (**b**), and 20,000× (**c**).

Figure 6a–c includes images obtained by scanning electron microscopy (SEM) for the Nb_2_O_5_ catalyst.

Figure 6a,b presents scanning electron microscopy (SEM) images of Nb_2_O_5_. The structure is characterized by the presence of dense tubes on the surface and well-defined niobium plates, accompanied by regions with three-dimensional aggregates (Figure 6a,b). Thinner and denser plates are observed (Figure 6c), distinct from each other, reflecting the variability in particle size distribution previously discussed. This combination of morphologies and dimensions is attributed to thermal control during processing, which promotes the formation of heterogeneous and well-organized textures [46,57].

**Figure 7:**
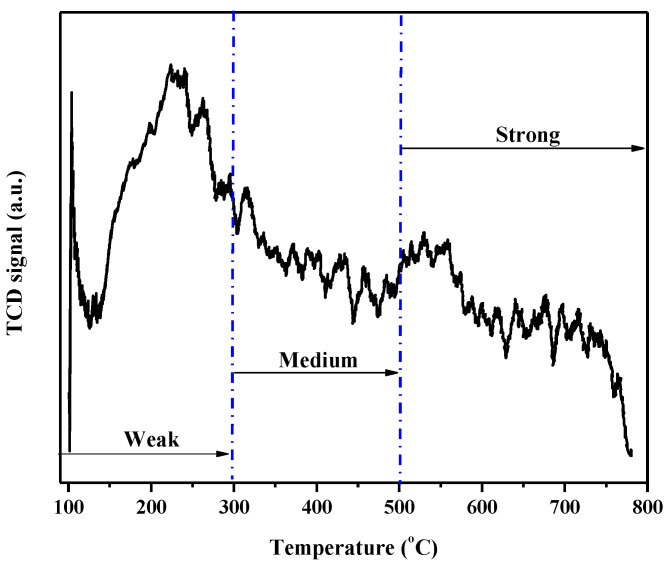
TPD-NH_3_ analysis of Nb_2_O_5_ catalyst.

Figure 7 shows the active acidic sites characterized using the temperature-programmed desorption (TPD) technique.

The TPD-NH_3_ analysis revealed the presence of different types of acidic sites in the Nb_2_O_5_ catalyst, classified according to the desorption temperature ranges. Up to 300 °C, weak acidic sites were identified, characterized by less intense interactions between ammonia molecules and the material’s surface. In the 300 to 400 °C range, moderate acidic sites predominated, with intermediate strength interactions. Above 500 °C, strong acidic sites were observed [58]. The analysis showed that the most significant quantity and intensity of acidic sites were found in the region corresponding to weak acidic sites, with a peak of the highest intensity around 200 °C. Despite being associated with sites of lower acidity strength, these conditions suggest a relevant catalytic behavior at temperatures typical of processes requiring acidity in severe environments [59].

### 2.2. Catalytic Tests

Table 4 presents the conversion and yield results of oils obtained using Nb_2_O_5_ as a catalyst and different feedstocks.

The results presented in Table 4 show significant variations in conversion and yield values depending on the feedstock used and the employed route (methanol and ethanol). Conversion refers to the fraction of triglycerides that have been transformed into esters, while yield expresses the final amount of biodiesel obtained relative to the initial oil mass. Thus, it is possible to achieve a high yield even with a relatively low conversion, as yield depends not only on the conversion of triglycerides but also on the recovery and purification of the final product.

The methanol-based route exhibited higher conversions than the ethanol-based route, reaching 96.43% for soybean and 78.34% for corn, while the ethanol-based route resulted in lower conversions of 21.55% and 13.32%. Regarding yield, the ethanol-based route provided slightly better results for soybean (74.63%) and residual feedstock (78.76%). In comparison, the methanol-based route achieved the highest yield for sunflower (84.13%) and similar yields for corn (methanol: 79.83% and ethanol: 79.82%). For residual feedstock, the ethanol-based route demonstrated the best overall performance, achieving a conversion of 69.74% and a yield of 78.76%, surpassing the methanol-based route.

The reduced surface area of Nb_2_O_5_ (1.30 m^2^/g) limits reagent accessibility to active sites, impacting its catalytic efficiency in heterogeneous reactions. Although it exhibits a high density of acidic sites (231.01 μmol NH_3_/m^2^) and significant total acidity (301.00 μmol NH_3_/g), its low pore volume (0.01 cm^3^/g) indicates a compact structure, restricting molecular diffusion and reducing ester conversion. Da Conceição et al. (2016) [35] reported that increasing the thermal treatment temperature reduces the surface area due to the compaction of the crystalline structure. This effect decreases the number of accessible acidic sites for catalysis but enhances structural stability.

The density values obtained for the biodiesel samples range from 904.6 kg/m^3^ to 951.8 kg/m^3^, which means they are outside the range established by the ASTM D4052 standard [60] (850–900 kg/m^3^). These higher values may indicate the influence of the fatty acid composition, such as a higher molecular weight and lower degree of unsaturation [61]. The acidity levels also exceed the established limit (0.50 mg KOH/g) for most tests, especially for the residual feedstock. The high acidity may be related to the presence of unconverted free fatty acids, indicating incomplete conversion.

#### 2.2.1. Effect of Feedstock

The fatty acid composition plays a crucial role in selecting the appropriate feedstock, as the physicochemical properties of biodiesel, such as viscosity, cetane number, and heat of combustion, are directly influenced by the structure of fatty acid esters. Oils rich in polyunsaturated acids contain carbon chains with multiple double bonds, which lower the cetane number and increase fluidity. In contrast, longer and more saturated chains enhance stability and combustion performance [30,62]. The choice of feedstock impacts factors such as cost, yield, composition, and purity of the final product, as different raw materials exhibit variations in free fatty acid content and oil compositions [63].

According to Table 4 and Table 5, soybean oil is characterized by a low content of saturated fatty acids and a moderate proportion of monounsaturated acids. These characteristics facilitated transesterification, optimizing interactions with the Brønsted and Lewis acid sites of Nb_2_O_5_. In contrast, corn and sunflower oils contain higher levels of polyunsaturated fatty acids, such as linoleic acid, and a lower proportion of monounsaturated acids. This composition may affect reaction kinetics by requiring more specific catalytic conditions, potentially reducing the efficiency of Nb_2_O_5_, particularly in standardized processes that do not account for these differences. Residual oil is distinguished by its greater compositional heterogeneity, with the presence of free and saturated fatty acids. These factors, along with possible impurities, may have limited the achieved conversions, as soap formation could occur, necessitating adjustments in catalytic conditions to mitigate these effects.

#### 2.2.2. Effect of the Route Used

Methanol and ethanol are the primary alcohols used in biodiesel production. Methanol is used due to its high reactivity in the triglyceride transesterification process. However, its production from fossil sources represents a limitation in terms of sustainability. Ethanol, in turn, stands out as a renewable alternative obtained from biological sources, such as sugarcane. In addition to being often more economical than methanol, ethanol has advantages such as low toxicity, greater safety in handling, and versatility of use [64,65].

The high conversion in the methyl route, mainly observed in soybean oil, is attributed to its homogeneous composition and low content of free fatty acids. Soybean oil has excellent miscibility with methanol, which results in high biodiesel yields. The ethyl conversion is reduced, probably due to the lower reactivity of ethanol and the difficulty of dissolving this alcohol in the lipid matrix.

Compared to soybean oil, residual oil exhibits reduced methyl conversion, which can be explained by the higher content of free fatty acids, which favor secondary reactions, such as saponification. The ethyl route presents superior performance due to the greater tolerance of Nb_2_O_5_ to conditions of high acidity, which is a constant in this type of oil [66].

For corn oil, the lower conversion in the methyl route can be attributed to the free fatty acid composition and a slight difference in the miscibility of the oil with alcohol [67]. The ethyl route, in turn, shows extremely low conversion, suggesting that Nb_2_O_5_ is not optimized for the chemical composition of corn oil in this route, possibly due to the limitations in the active sites for reactions involving longer-chain alcohols and the reaction conditions used. Regarding sunflower oil, which has a high proportion of polyunsaturated fatty acids, the methyl route resulted in moderate conversion, likely due to challenges associated with transesterification under standard conditions and potential side reactions leading to undesirable byproducts. The ethyl route, however, showed even lower conversion, indicating that the interaction between Nb_2_O_5_ and ethanol was not as effective for this feedstock [68].

#### 2.2.3. Catalyst Reuse

The catalytic stability of Nb_2_O_5_ was evaluated in the transesterification of soybean oil with methanol under the same conditions as the initial tests (AOR = 15:1, 4 wt% catalyst, 200 °C, and 1 h). Table 5 presents the percentage distribution of the compounds formed over five reuse cycles.

The ester percentages obtained demonstrate a considerable reduction in the efficiency of Nb_2_O_5_ over five reuse cycles when compared to the initial conversion of 96.43% achieved with the fresh catalyst. In the first cycle, the conversion to esters dropped sharply to only 10%, indicating a significant decline in catalytic activity upon the first reuse. Although a moderate increase was observed in the third cycle, reaching 21%, subsequent cycles showed lower values, with 15% in the fourth cycle and 19% in the fifth.

The variation in conversion may result from uneven removal of adsorbed species, affecting the exposure of active sites. The increase in the third cycle suggests partial reactivation of acidic sites due to the gradual release of residues. The subsequent decline indicates progressive deactivation by organic deposit poisoning. The yield of methyl esters remained stable (79.20–83.35%), suggesting that, despite the conversion variation, the final product’s formation was not significantly compromised.

The reduction in catalytic efficiency may be attributed to the accumulation of byproducts, such as free fatty acids, or the possible partial deactivation of active sites. However, the structural integrity of Nb_2_O_5_ appears to have remained intact. The results do not indicate abrupt changes in performance, suggesting a nearly linear decline in catalytic activity over repeated cycles.

##### Characterization of the Reused Catalyst

**Figure 8:**
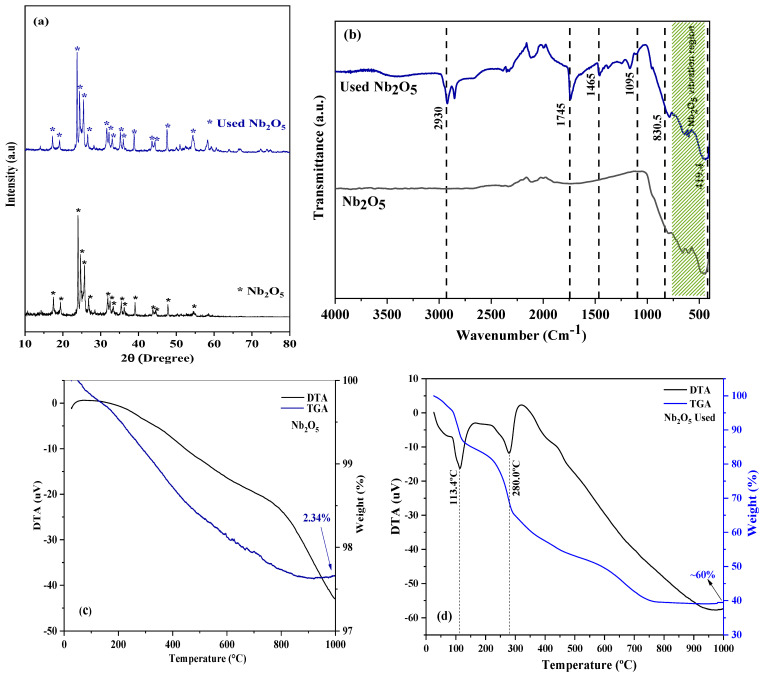
(**a**) X-ray diffraction (XRD) patterns, (**b**) FTIR spectra of Nb_2_O_5_ in both fresh and reused conditions, and TGA/DTA curves of (**c**) fresh and (**d**) reused Nb_2_O_5_.

FTIR and XRD analyses were conducted to evaluate the structural and chemical stability of Nb_2_O_5_ after the reuse cycles. Figure 8 illustrates the (a) X-ray diffraction (XRD) patterns and (b) FTIR spectra of Nb_2_O_5_ in both fresh and reused conditions.

The analysis of Figure 8a showed that the crystalline phase of Nb_2_O_5_ remained unchanged over the five reuse cycles, indicating that the crystalline structure of the catalyst maintained its integrity. The analysis of the FTIR spectra (Figure 8b) revealed significant changes on the surface of the Nb_2_O_5_ catalyst after reuse. The characteristic range of 450 to 800 cm^−1^, associated with the Nb_2_O_5_ structure, was preserved in the used catalyst, indicating the retention of the material’s structural integrity. However, the appearance of new bands suggests the accumulation of intermediate and adsorbed products on the surface. The band at 2930 cm^−1^ is attributed to the symmetric stretching of CH_3_, while the band at 1745 cm^−1^ corresponds to the stretching of C=O in esters, indicating the adsorption of product molecules on the catalyst surface. The bands at 1465 cm^−1^, related to the asymmetric deformation of CH_3_, and at 1095 cm^−1^, assigned to stretching C-O groups in esters, support the retention of organic compounds in the material’s structure. These results are consistent with previous studies that link the reduction in catalytic activity to blocking active sites by adsorbed species, such as intermediates or reaction products [24,35].

The TGA and DTA analyses (Figure 8d) revealed two distinct thermal events centered at 113.4 °C and 280 °C, associated with the decomposition of organic residues accumulated over successive reuse cycles. A total mass loss of approximately 60% was observed, primarily due to the desorption and decomposition of organic material retained on the catalyst surface. The FTIR analysis corroborates the findings from the TGA and DTA curves, reinforcing the presence of organic residues accumulated during catalyst reuse. This result demonstrates that solid-base catalysts undergo neutralization in the presence of free fatty acids [14].

## 3. Experimental Section

### 3.1. Materials

In this study, the following materials were used: niobium pentoxide (Nb_2_O_5_), purchased from Companhia Brasileira de Metalurgia e Mineração (CBMM) (Araxá, Brazil), supplied in powder form with a high degree of purity (99%). Various vegetable oils were used as raw materials for catalytic evaluation, including soybean oil (Soya, Stockholm, Sweden), sunflower oil (Liza, Aachen, Germany), corn oil (Liza) acquired from local commerce, and residual oil. Methanol (CH_3_OH, 99%, Dinâmica—Serra, Brasil) and n-hexane (C_6_H_14_, 99%, Neon—Sao Paulo, Brasil) were also used. The residual oil was filtered before experiments using filter paper with a diameter of 15.00 ± 0.15 cm to remove particulate material.

The chemical composition of the oils is presented in Table 6, including their acidity.

The selection of vegetable oils for this work was motivated by the greater availability of crops that these oils offer in our locality due to their high triglyceride content. The production of biodiesel from these raw materials is comparatively simpler and more efficient, making using these sources a viable option.

### 3.2. Methods

#### 3.2.1. Catalyst Preparation

The only procedure employed involved sieving the material using a 200-mesh screen to homogenize the particles for application in catalytic tests.

#### 3.2.2. Physico-Chemical Characterization

The crystalline structure of the Nb_2_O_5_ catalyst was analyzed by X-ray diffraction (XRD) using a BRUKER D2 PHASER diffractometer (BRUKER, Billerica, MA, USA) with Cu-Kα radiation (*λ* = 1.54056 Å), operating at 30 kV and 10 mA. Data collection was performed over a 2θ range of 10° to 70°, with an angular step of 0.016° and a counting time of 1.000 s per step. Phase identification and crystallinity determination were conducted using the ICDD PDF-2 (2019) database and processed with DiffracPlus Suite Eva software V6.0.

Thermal stability and decomposition behavior were assessed by thermogravimetric (TGA) and differential thermal analysis (DTA) using a Shimadzu TA-60H system (Shimadzu, Kyoto, Japan), under an air atmosphere (100 mL/min) with a heating rate of 12.5 °C/min up to 1000 °C. The elemental composition was analyzed through energy-dispersive X-ray fluorescence (EDX) spectroscopy using a Shimadzu EDX-720 system. Particle size distribution was evaluated using the laser diffraction technique with an SZ-100 series nanoparticle analyzer HORIBA Scientific (Kyoto, Japan).

Fourier transform infrared (FTIR) spectroscopy was performed using a BRUKER Vertex 70 spectrometer. Spectra were recorded in transmittance mode over a wavenumber range of 4000 to 200 cm^−1^, with a total of 64 scans.

The textural properties of the Nb_2_O_5_ catalyst were evaluated through nitrogen adsorption–desorption analysis using a Micromeritics AutosorbIQ system (Micromeritics, Norcross, GA, USA). The specific surface area was determined by the Brunauer–Emmett–Teller (BET) method, while pore volume and diameter were assessed using the Barrett–Joyner–Halenda (BJH) method.

The actual density of the Nb_2_O_5_ catalyst was determined using a Quantachrome Upyc 1200e v5.04 pycnometer (Quantachrome, Boynton Beach, FL, USA) with helium gas. The morphological characteristics of the catalyst were examined using scanning electron microscopy (SEM) with a Tescan Vega3 microscope (Tescan, Brno, Czechia).

The acidity of the Nb_2_O_5_ catalyst was evaluated by temperature-programmed desorption of ammonia (TPD-NH_3_) using the SAMP3 multipurpose analysis system (SAMP, Bologna, Italy). Prior to analysis, approximately 100 mg of sample was pretreated at 400 °C under a helium flow (30 mL/min). After cooling to 100 °C, the sample was exposed to an ammonia flow for 45 min to allow chemical adsorption. Excess NH_3_ was subsequently removed by maintaining the sample at 100 °C for 1 h under a continuous helium flow. The desorption thermograms were recorded during heating from 100 °C to 800 °C at a rate of 10 °C/min under a helium atmosphere (30 mL/min).

The composition of the feedstock and the percentage of ethyl esters were analyzed using gas chromatography on a VARIAN 450c chromatograph (VARIAN, Palo Alto, CA, USA), equipped with a flame ionization detector. A capillary column (Varian Ultimetal ’Select Biodiesel Glycerides RG’; dimensions: 15 m × 0.32 mm × 0.45 mm) served as the stationary phase. The initial injection and oven temperatures were set at 100 °C and 180 °C, with the detector maintained at 380 °C.

The practical yield was calculated as the mass of biodiesel obtained in relation to the mass of the raw material used, as shown in Equation (1).

(1) $$yield\;\left(\%\right)=\left(\frac{Mass\;of\;biodiesel\;obtained}{Mass\;of\;raw\;material}\right)\times 100$$

The density was determined following the ASTM D4052 standard using an Anton Paar DMA 35 Version 3 densimeter (Anton Paar, Graz, Austria). The acid value (official AOCS method, Cd 3d-63) [69] was used to characterize both the feedstocks and the products resulting from the catalytic tests.

### 3.3. Catalytic Test

The biodiesel produced was washed with distilled water at ~70 °C and dried in an oven at 110 °C for 30 min with manual stirring at 5 min intervals. The performance of Nb_2_O_5_ catalysts was assessed in biodiesel synthesis through transesterification and esterification reactions, using soybean oil, waste oil, corn oil, and sunflower oil as feedstocks. Catalytic tests were performed in a stainless-steel reactor (Parr 4848, 100 mL capacity, Hillsboro, OR, USA) equipped with a mechanical stirrer, time and temperature controller, and pressure indicator. The experimental conditions were set as follows: reaction temperature of 200 °C, 30 g of oil, and 4% catalyst (based on oil mass), with stirring at 600 rpm for 1 h. Following the reactions, the catalysts were separated from the products using centrifugation (3500 rpm), then purified with n-hexane, and dried in an oven at 110 °C for 24 h. The biodiesel produced was further washed with distilled water at around 70 °C and dried in an oven at 110 °C for 30 min, with manual stirring every 5 min.

#### Catalyst Reuse

Following the reaction, the catalysts were retrieved, purified with n-hexane, and then dried in an oven at 110 °C for 24 h. Reusability tests were performed under the same conditional conditions as the catalytic test results.

## 4. Conclusions

Nb_2_O_5_, in its commercial monoclinic crystalline structure, has proven to be an effective and structurally stable catalyst for biodiesel production through the transesterification of vegetable oils, such as soybean oil, using methanol. X-ray diffraction (XRD) analyses confirmed that the catalyst’s crystalline phase was maintained after successive reuse cycles. However, FTIR spectra indicated the adsorption of byproducts on the material’s surface, contributing to the gradual reduction of its catalytic activity. Thermogravimetric (TGA) and differential thermal analysis (DTA) further supported this observation, revealing two distinct thermal events associated with the decomposition of organic residues accumulated over successive reuse cycles.

The catalytic performance varied according to the feedstock and alcohol used. While Nb_2_O_5_ exhibited high conversion rates in the methanol-based transesterification of soybean oil, its efficiency was notably lower for ethanol-based reactions and other feedstocks. These differences are attributed to variations in free fatty acid content and composition, which directly impact catalyst deactivation and reactivity.

In the initial tests, Nb_2_O_5_ achieved a significant conversion of 96.43% to methyl esters, demonstrating its high efficiency under specific reaction conditions (AOR = 15:1, 4% catalyst, 200 °C, one h). After five reuse cycles, a significant reduction in conversion was observed, reaching 18.5% in the final cycle, likely due to the partial deactivation of the active sites blocked by intermediates or adsorbed byproducts.

Nonetheless, the catalyst’s structural stability, coupled with the retention of a portion of its catalytic activity, highlights its potential for applications in industrial systems and provides strategies that are implemented to minimize deactivation. The results of this study support the viability of using Nb_2_O_5_ as a heterogeneous catalyst in transesterification processes, reinforcing its applicability in the sustainable production of biodiesel from low-cost and readily available raw materials.

## Figures and Tables

**Table 1:** Biodiesel conversion using Nb_2_O_5_ as a catalyst.

Catalyst	Crystal Phase	Feedstock	Route	Reaction Conditions	Conversion	Ref.
H_4_PNbW_11_O_40_/WO_3_–Nb_2_O_5_	-	Oleic acid	Ethanol	AOR * = 15:1, 11.11% wt ** of catalyst, 99.85 °C, 8 h	70.0%	Katada, Hatanaka [33]
25wt%TPA/Nb_2_O_5_	TT-Nb_2_O_5_	Sunflower oil and Palmitic acid	Methanol	AOR = 13.7:1, 15% wt of catalyst 65 °C, 4 h	97.3% 99.1%	Srilatha, Lingaiah [34]
MCM-41-Nb-8	-	Sunflower oil	Methanol	AOR = 12:1, 7.5% wt of catalyst 200 °C, 4 h	95.0%	García et al. [14]
Nb_2_O_5_/SO_4_	TT-Nb_2_O_5_	Palm oil	Ethanol	AOR = 120:1, 30% wt of catalyst, 250 °C, 4 h	99.2%	da Conceição, Carneiro [35]
2Nb_2_O_5_/SBA-15	Amorphous	Propanoic acid	Methanol	AOR = 15:1, 1% wt of catalyst, 120 °C, 4 h	92.0%	Silva, Wilson [36]
30MoO_3_/Nb_2_O_5_	TT-Nb_2_O_5_	Waste oil	Methanol	AOR = 30:1, 5% wt of catalyst, 145 °C, 2.5 h	94.3%	de Brito, Gonçalves [37]

* alcohol:oil ratio (AOR); ** (% wt) weight by mass.

**Table 2:** Percent composition of the oxides present in the Nb_2_O_5_ catalyst determined by X-ray fluorescence (EDX).

Catalyst	Oxides Presents			
	NbO	SiO_2_	F_2_O_3_	Others
Nb_2_O_5_	96.71	2.95	0.14	0.2

**Table 3:** Structural and acidic properties of the Nb_2_O_5_ catalyst.

Catalyst	Surface Area (m^2^ g^−1^)	Pore Volume (cm^3^ g^−1^)	Density (g/cm^3^)	Total Acidic Sites (μmoles NH_3_ g^−1^)	μmol of NH_3_ m^−2^
Nb_2_O_5_	1.30	0.01	4.64	301.00	231.01

**Table 4:** Conversion, practical yield, density, and acidity.

Feedstock	Route	Conversion (%)	Yield (%)	Density (Kg·m^−3^)	Acidity (mg KOH·g^−1^)
Soybean	Methanol	96.43 ± 0.14	65.43	908.3	0.82 ± 0.07
	Ethanol	21.55 ± 8.40	74.63	921.1	0.82 ± 0.07
Residual	Methanol	77.05 ± 6.11	74.03	951.8	5.73 ± 0.07
	Ethanol	69.74 ± 9.52	78.76	949.6	6.29 ± 0.07
Corn	Methanol	78.34 ± 13.47	79.83	910.9	1.37 ± 0.07
	Ethanol	13.32 ± 4.05	79.82	904.6	0.55 ± 0.00
Sunflower	Methanol	63.04 ± 11.47	84.13	912.4	1.37 ± 0.07
	Ethanol	33.37 ± 9.66	75.26	918.1	1.09 ± 0.00

**Table 5:** Evaluation of the catalytic stability of Nb_2_O_5_.

Cycle	Conversion (%)	Yield (%)
1	10 ± 1.72	79.20
2	19 ± 4.72	83.35
3	21 ± 8.85	80.18
4	15 ± 5.33	81.36
5	18 ± 0.69	80.60

**Table 6:** Chemical composition of the oils.

Feedstocks	Main Fatty Acids					Acidity (mg KOH·g^−1^)
	Palmitic C_16:0_	Stearic C_18:0_	Oleic C_18:1_	Linoleic C_18:2_	Linolenic C_18:3_>	
Soybean	1%	4%	29%	63%	3%	0.00
Residual	12%	4%	18%	54%	12%	8.19
Corn	2%	5%	31%	60%	2%	0.00
Sunflower	2%	1%	18%	77%	2%	0.00

## Data Availability

The original contributions presented in this study are included in the article. Further inquiries can be directed to the corresponding authors.
